# Status of water, sanitation and hygiene services for childbirth and newborn care in seven countries in East Asia and the Pacific

**DOI:** 10.7189/jogh.09.020430

**Published:** 2019-12

**Authors:** Priya Mannava, John CS Murray, Rokho Kim, Howard L Sobel

**Affiliations:** 1 Maternal, Child Health and Quality Safety, World Health Organization Western Pacific Regional Office, United Nations Avenue, Manila, Philippines; 2 Health and the Environment, World Health Organization Western Pacific Regional Office, United Nations Avenue, Manila, Philippines

## Abstract

**Background:**

Water, sanitation and hygiene (WASH) services are critical to providing quality maternal and neonatal care in health facilities. This study aimed to investigate availability of WASH policies, standards, and services for childbirth and newborn care in hospitals in East Asia and the Pacific.

**Methods:**

Descriptive analysis of survey data and observations of water, sanitation and hygiene services in maternity and neonatal care rooms and of deliveries in 147 hospitals in Cambodia, Lao People’s Democratic Republic, Mongolia, Papua New Guinea, Philippines, Solomon Islands, and Viet Nam. The main outcome measures were availability of national policies and standards; availability of water, sanitation, and hygiene services in maternity rooms and neonatal care units; and practice of hygiene at childbirth.

**Results:**

Three of seven countries had national WASH policies and three had standards for health facilities. Seventy-seven percent of hospitals had a sink with water and soap or alcohol hand rub in delivery rooms, 78% in neonatal care rooms and 42% in postnatal care rooms. Only 44% of hospitals had clean sinks with water, soap and hand drying methods in the delivery room, 40% in neonatal care units and 10% in postnatal care rooms. Flush toilets were available in or next to delivery rooms in 60% and neonatal care units in 50% of 10 hospitals with data. Countries with WASH standards had a higher proportion of hospitals with water and hand hygiene services. Appropriate hygiene was practiced by health workers in 65% of 371 deliveries observed, and more likely in delivery rooms with a sink, water and soap.

**Conclusions:**

Coverage of WASH services for maternal and newborn care must be improved to reduce risks of maternal and newborn morbidity and mortality.

An estimated 139 000 newborns (0-27 days) died in East Asia and the Pacific in 2018, representing 49% of deaths in children under the age of five years [1]. Up to 20% of these deaths were from preventable infections: sepsis, pneumonia, diarrhoea, tetanus and meningitis [2]. Neonatal infections are acquired during passage in the birth canal or exposure to unhygienic care practices and environments [3]. The risk of infection among hospital-born babies in low- and middle-income countries (LMICs) is 3-20 times higher compared to in high-income countries, adding considerable costs to systems least able to bear them [3]. As over 95% of births in East Asia and the Pacific are facility-based, infection prevention and control (IPC) practices in health facilities are critical to reducing morbidity and mortality.

The importance of hygiene practices during childbirth and the postpartum period is well recognized [3-6]. Practices such as hand-washing by birth attendants, clean birthing surfaces and clean cord cutting are associated with reductions in all-cause sepsis and tetanus neonatal mortality [4]. These essential practices require adequate water, sanitation and hygiene (WASH) services.

In early 2019, the World Health Organization (WHO) and United Nations Children’s Fund (UNICEF) reported that globally 26% of health facilities did not have access to an improved water source on the premises, 16% had no hand hygiene services at points of care, and 21% did not have sanitation services [7]. In Kenya, Rwanda, Tanzania, and Uganda, less than 30% of 1333 facilities surveyed had piped water in the delivery room and functioning toilets or latrines for clients [8]. In Tanzania, only 24% of delivery rooms had soap for handwashing available [9]. Improving coverage of WASH services requires action in several areas including development of national policies and standards and additional data on the status of WASH in health facilities [7].

Despite the burden of neonatal infections in East Asia and the Pacific, data on WASH services for childbirth and newborn care in health facilities are limited. The objectives of this study were to: (1) determine availability of national policies and standards on WASH services in health facilities and whether these specify services for childbirth and newborn care, (2) describe the status of WASH services in maternity rooms and neonatal care units (NCUs), and hygienic practices at the time of childbirth, and (3) determine if national policies and standards are associated with availability of water and hygiene services in seven countries in the region.

## METHODS

### Sample and data sources

Seven countries with the highest burden of neonatal mortality and accounting for over 97% of neonatal deaths in East Asia and the Pacific were selected for this study: Cambodia, Lao People’s Democratic Republic (Lao PDR), Mongolia, Papua New Guinea, Philippines, Solomon Islands, and Viet Nam [1].

A short survey for Ministries of Health was developed asking: (1) whether the country had a national policy and/or standards on WASH service requirements in health facilities, and if yes, (2) if these specified services for maternity and neonatal care, and (3) for a copy (in English or the local language). The survey was sent to Ministries of Health in the seven countries through the respective WHO Representative Offices. Where applicable, country responses were validated by reviewing national documents.

To determine status of water and hygiene services and hygienic practices at childbirth, we undertook secondary analyses of data from national implementation reviews of early essential newborn care (EENC) conducted in the seven countries in 2016-17. Data were also available from an eighth country but excluded due to the very small sample size of hospitals included in the implementation review. All the seven countries have introduced EENC, a package of evidence-based practices known to reduce preventable causes of neonatal mortality [10], in health facilities with at least 50 deliveries per year beginning first with hospitals. Implementation of EENC involves clinical coaching for staff on childbirth and immediate newborn care, formation of hospital EENC teams who monitor quality of care, and use of data to inform improvements in hospital policies and protocols, clinical practices and work environments, including WASH services [10].

Annual or biennial national implementation reviews assess childbirth and newborn care in a sample of hospitals that have introduced EENC [11]. For these reviews, the sampling frame is all hospitals implementing EENC, with random selection of three national or regional tertiary hospitals, 4-12 provincial hospitals and 2-4 district hospitals within each of the provinces sampled. Countries with lower rates of EENC implementation have smaller sample sizes. Larger countries such as Philippines and Viet Nam conduct stratified sampling in the main administrative regions of the country and have larger sample sizes.

During the national implementation reviews in the seven countries, trained assessors used standard methods and tools adopted for local use to assess care in each sampled hospital over 1-2 days [11]. Postpartum mothers were interviewed and their patient charts reviewed, and deliveries, environmental hygiene and availability of medicines and essential supplies observed. For the purposes of this study, we analyzed data collected from observations of environmental hygiene and deliveries.

Environmental hygiene was observed in delivery and postnatal care rooms and NCUs using a standard checklist [11]. In each room, assessors observed: cleanliness of sinks and availability of running water, soap, hand drying methods, and alcohol-based hand rub at the time of the assessment and recorded findings in the checklists. Assessors observed deliveries during the assessment period at each hospital, and recorded delivery practices using a standard checklist based on EENC standards [11]. Key hygiene practices analyzed for this study were appropriately timed hand washing prior to touching delivery area surfaces and equipment and immediately prior to gloving for delivery, and hygienic handling of the umbilical cord with uncontaminated sterile gloves.

EENC teams in a subset of hospitals in the seven countries conduct routine quality improvement assessments to self-monitor quality of care using a standard tool [12]. Data are used to identify gaps in care and take action for improvement. Similar data to the national implementation reviews are collected, with observations of cleanliness and functionality of sanitation facilities added to environmental hygiene assessments. Data from quality improvement assessments for the years 2015-2016 were analyzed to determine availability of sanitation facilities.

### Variable definitions

Hospitals were divided into tertiary and first-level referral categories based on capacity to manage complications of delivery and provide advanced neonatal care. First-level referral hospitals can manage delivery complications, preterm deliveries and common complications of prematurity and advanced resuscitation. Tertiary hospitals offer these same services in addition to advanced neonatal care and serve as teaching hospitals. National policies were defined as providing general direction on planning, management and implementation of WASH services at the highest level (they can be specific for health facilities or general WASH policies that include health facilities), and standards defined as the minimum level of WASH services required in health facilities (specific for a category of health facility or across several health facility categories).

Variables on access to basic water supply, sanitation and hygiene services were constructed using modified definitions proposed by the WHO/UNICEF Joint Monitoring Programme for Water Supply and Sanitation (JMP) (**Table 1**) [13]. JMP defines “access” to basic water supply and sanitation as availability on the premises of the health care facility. We reasoned however, that for IPC and patient comfort, water was required in and sanitation in or adjacent to the rooms where care was given. Where hospitals had more than one delivery, postnatal or neonatal care room, we defined access as availability in all the rooms. Sinks and toilets/latrines were deemed clean if no stains, visible waste, dirt or items (such as medical instruments or supplies) were present.

**Table 1:** Definitions used for WASH services in delivery and postnatal care rooms and NCUs

Water supply	Sanitation	Hygiene
Basic: Water is available in the room from an improved source* at the time of the assessment	Basic: Toilets or latrines^†^ that are functional^‡^ are available in or adjacent to the room at the time of the assessment.	Basic: Hand hygiene stations including a sink with water and soap, or alcohol-based hand rubs, are available in the room at the time of the assessment.
	Clean: Toilets or latrines do not have stains, visible waste, dirt or items inside.	Adequate: All hand hygiene stations have sinks that are clean (no stains, visible waste, dirt or items inside) with running water, soap and a hand drying method^§^; or alcohol-based hand rub available in the room at the time of the assessment

NCU – neonatal care unit

*Improved water source: piped water, stored water with or without a tap

†Toilets and latrines include: flush/pour flush toilets, ventilated improved pit latrines, pit latrines with slab, and composting toilets.

‡Functional toilets or latrines: Hole or pit should not be blocked, water is available for flush/pour flush toilets, and no cracks or leaks in toilet structure. Toilet stall walls should have no major holes and a door which can be locked from the inside during use.

§Hand drying methods: single-use towels, functioning electric hand driers, or reusable sterile towels.

In countries where basic services are met, the JMP encourages advanced service levels to be defined at the national level. We constructed an additional variable “adequate hygiene” based on WHO standards for improving maternal and newborn care in health facilities [14] and reflecting all conditions required to ensure hand hygiene is strictly followed by health workers (**Table 1**).

### Analysis

Data for the WASH and delivery practice variables were extracted from the national implementation review and quality improvement assessment (for data on sanitation facilities) data sets. Descriptive statistics were used to summarize data on availability of national policies and standards, WASH services in hospitals by room type and hospital level, and hygienic delivery practices by hospital level. Analyses on WASH services and delivery practices were anonymized to be non-country specific. Differences in services between hospital levels were compared using χ-2 tests. Associations between national policies and standards with availability of water and hand hygiene services in maternity and neonatal care rooms in health facilities were determined in bivariate logistic regression. Data were analyzed using the Intercooled Stata 13.0 statistical package (StataCorp, College Station, TX, USA).

### Ethical procedures and approvals

As Ministries of Health undertook the implementation reviews as part of monitoring of national EENC programmes, and findings intended solely to inform programme planning, ethical review was not deemed necessary in country. Assessments did not influence the time or place of deliveries, staff responsible for care, require deviations from accepted clinical practices, nor impose significant additional burden on patients, families or staff. Informed verbal consent was secured prior to delivery observations, and no personal identifiers used. Consent was obtained from management of each hospital assessed. This study was also exempt from requiring review by the WHO Regional Office for the Western Pacific Ethical Review Committee on the basis that secondary analysis of observational data was undertaken with no patient identifiers used.

## RESULTS

### National policies and standards on WASH services in health facilities

Lao PDR, Papua New Guinea and Solomon Islands had a national WASH policy covering service provision in health facilities at the time of the survey (**Table 2**). No policies specified requirements for maternal and neonatal care. China, Mongolia, Philippines and Solomon Islands had national standards for WASH services in health facilities - one of which outlined water and hand hygiene requirements in maternity rooms and NCUs, as well as sanitation facilities for pregnant women.

**Table 2:** Status of national policies, standards or guidelines on WASH services in health facilities, seven countries, 2017

Country	National policy (Yes, Draft, No)	Policy specifies services required in maternity rooms and NCUs (Yes or No)	Standards or guidelines (Yes, Draft, No)	Standards or guidelines specify services required in maternity rooms and NCUs (Yes or No)
Cambodia	No	Not applicable	Draft	No
Lao PDR	Yes	No	Draft	No
Mongolia	No	Not applicable	Yes	Yes
PNG	Yes	No	No	Not applicable
Philippines	No	Not applicable	Yes	No
Solomon Islands	Yes	No	Yes	No
Viet Nam	No	Not applicable	Draft	Yes

NCU – neonatal care units

### WASH services and hygienic delivery practices

A total of 147 hospitals, representing around 12% of hospitals with more than 50 deliveries per year in the seven countries [15], were assessed for childbirth and newborn care as part of national implementation reviews in 2016-17 in the seven countries (**Table 3**).

**Table 3:** Characteristics of hospitals assessed for WASH services in seven countries, 2015-2017

Country	Hospitals assessed, 2016-17*, n (%)	Annual live births in hospitals assessed, 2016, median (range)	Tertiary hospitals assessed, 2016-17*, n (%)	Hospitals with delivery observations, 2016-17*n (%)	Tertiary hospitals with delivery observations,* n (%)	Hospitals assessed for sanitation facilities, 2015-16†, n (%)	Tertiary hospitals assessed for sanitation facilities, 2015-16†, % (n)
Cambodia	15 (10.2)	1415 (212-7634)	3 (10.4)	8 (7.9)	2 (8.0)	0 (0.0)	0.0 (0)
Laos	18 (12.2)	1892‡ (1038-13869)	4 (13.8)	10 (9.9)	3 (12.0)	4 (40.0)	80 (4)
Mongolia	25 (17.)	1724 (449-12058)	4 (13.8)	22 (21.8)	4 (16.0)	0 (0.0)	0.0 (0)
Papua New Guinea	6 (4.1)	1745 (700-12613)	1 (3.4)	5 (5.0)	1 (4.0)	6 (60.0)	20 (1)
Philippines	28 (19.0)	2868§ (159-15093)	13 (44.8)	27 (26.7)	13 (52.0)	0 (0.0)	0.0 (0)
Solomon Islands	7 (4.8)	644‖ (212-5809)	1 (3.4)	2 (2.0)	0 (0.0)	0 (0.0)	0.0 (0)
Viet Nam	48 (32.7)	2045¶ (614-65 119)	3 (10.4)	27 (26.7)	3 (12.0)	0 (0.0)	0.0 (0)
**Total**	**147 (100.0)**	**1971 (159-65 119)**	**29 (100.0)**	**101 (100.0)**	**25 (100.0)**	**10 (100.0)**	**100.0 (5)**

*Data from national EENC implementation reviews.

†Data from hospital quality improvement self-assessments.

‡Data available from 17 hospitals.

§Data available from 27 hospitals.

‖Data available from 6 hospitals.

¶Data on live births per hospital were available for three tertiary hospitals. Figures provided are estimated for the 48 hospitals using Ministry of Health data on total live births per province.

Two first-level referral hospitals did not have postnatal care rooms, whilst two tertiary hospitals and 30 first-level referral hospitals did not have NCUs. In tertiary hospitals, the median number of delivery rooms was 3 (range: 1-11), postnatal care rooms 5 (range: 0-128), and NCU rooms 3 (range: 0-32). First-level referral hospitals had a median of 2 delivery (range: 1-5), 4 postnatal care (range: 0-460), and 1 NCU (range: 0-13) rooms. The median number of annual live births across the hospitals assessed was 1972 (range 159-65 119).

Assessors observed a total of 371 deliveries in 101 of the 147 hospitals (between 1 and 10 deliveries at each hospital). Data from quality improvement assessments on sanitation services were available from 10 hospitals in Lao PDR, and Papua New Guinea for delivery rooms and NCUs only.

#### Access to WASH services

All hospitals had access to basic water supply – piped water – during the assessment period. However, only 71% (104/147) of hospitals had water supply in delivery rooms, 71% (82/115) in NCUs and 32% (46/143) in postnatal care rooms. Differences between hospital level were not significant.

Seventy-seven percent of hospitals (114/147) had basic hygiene services – a sink with water and soap or alcohol hand rub – in delivery rooms, 78% (89/114) in NCUs, and 42% (58/138) in postnatal care rooms, with non-significant differences by hospital level. A higher percentage of hospitals had alcohol hand rub compared to a sink with water and soap in delivery rooms (68% vs 65%, *P* = 0.46), postnatal care rooms (34% vs 18%, *P* = 0.002) and NCUs (71% vs 64%, *P* = 0.21).

**Figure 1:**
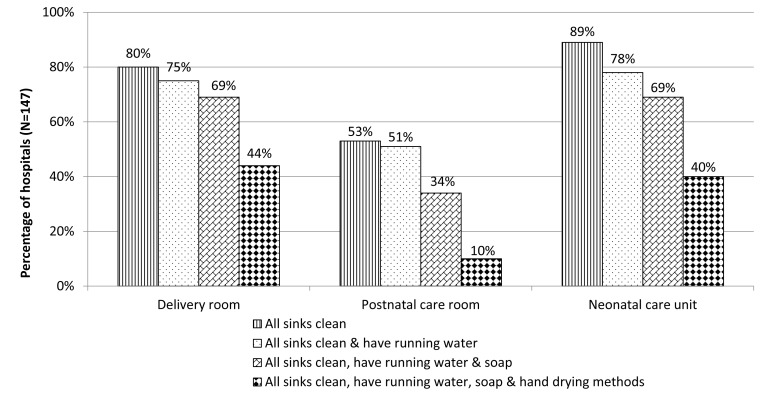
Availability of hand washing materials at hand hygiene stations in delivery rooms, postnatal care rooms and neonatal care units in 147 hospitals, 7 countries, 2016-17.

Seventy-two percent (106/147) of hospitals had clean sinks with running water, soap and hand drying methods or alcohol hand rub (adequate hygiene services) in delivery rooms, 73% (83/113) in NCUs and 36% (51/140) in postnatal care rooms. Differences between levels were only significant for NCUs, with 89% of tertiary hospitals providing adequate hygiene services compared to 69% of first-level referral hospitals (*P* = 0.04). While 71% of hospitals had piped water in delivery rooms, only 44% (64/147) had clean sinks with water, soap and availability of hand drying methods. The same trend was found for postnatal wards and NCUs (**Figure 1**).

National WASH policies were not significantly associated with increased likelihood of any service in hospitals. Countries with national standards were more likely to have all WASH services including piped water supply (odds ratio OR = 4.6, 95% confidence interval (CI) = 2.0-10.4), basic hygiene (OR = 2.6, 95% CI 1.3-5.2), and adequate hygiene (OR = 2.6, 95% CI 1.2-5.4) (**Table 4**).

**Table 4:** Associations between national policies and standards on WASH in health facilities and availability of services, assessments from 147 hospitals in 7 countries, 2016-17

Service*	Hospitals with service in countries with a policy n (%) (N = 31)	Hospitals with service in countries with no policy n (%) (N = 116)	Odds ratio (95% CI†)	*P* value	Hospitals with service in countries with standards n (%) (N = 60)	Hospitals with service in countries with no standards n (%) (N = 87)	Odds ratio (95% CI)	*P* value
Alcohol-based hand rub	3 (9.7)	36 (31.0)	0.2 (0.1, 0.8)	0.03	22 (36.7)	17 (19.5)	2.4 (1.1, 5.0)	0.022
Piped water supply	9 (29.0)	26 (22.4)	1.4 (0.6, 3.5)	0.44	24 (40.0)	11 (12.6)	4.6 (2.0, 10.4)	<0.001
At least one sink with water & soap	1 (3.2)	15 (12.9)	0.2 (0.0, 1.8)	0.16	12 (20.0)	4 (4.6)	5.2 (1.6, 17.0)	0.007
All sinks clean	5 (16.0)	57 (49.1)	0.2 (0.1, 0.6)	0.02	32 (53.3)	30 (34.5)	2.2 (1.1, 4.3)	0.024
All sinks clean & have running water	3 (9.7)	51 (44.0)	0.1 (0.0, 0.5)	0.02	29 (48.3)	25 (28.7)	2.3 (1.2, 4.6)	0.016
All sinks clean, having running water & soap	1 (3.2)	33 (28.5)	0.1 (0.1, 0.6)	0.02	20 (30.3)	14 (16.1)	2.6 (1.2, 5.7)	0.008
All sinks clean, having running water, soap, & hand drying methods	0 (0.0)	5 (4.3)	Not determined	Not applicable†	6 (9.1)	0 (0.0)	Not determined†	Not applicable
Basic hygiene (Sink with water & soap, or alcohol-based hand rubs)	4 (12.9)	42 (36.2)	0.3 (0.1, 0.8)	0.02	26 (43.3)	20 (23.0)	2.6 (1.3, 5.2)	0.010
Adequate hygiene (Clean sinks with running water, soap & hand drying methods, or alcohol-based hand rubs)	3 (9.7)	37 (31.9)	0.2 (0.1, 0.08)	0.02	23 (38.3)	17 (19.5)	2.6 (1.2, 5.4)	0.013

CI – confidence interval, NCU – neonatal care unit

*Measures availability of services in all delivery and postnatal care rooms and neonatal care units of the hospital.

†Not determined because in countries with policies and in countries with no standards, no sampled hospitals had the service in all delivery and postnatal care rooms and NCUs.

Of the 10 hospitals with data on sanitation services, 60% offered basic sanitation – flush toilets – for patients in or adjacent to the delivery room and next to the NCU. Fifty percent of hospitals had clean toilets for delivery rooms and 30% for NCU rooms. Compared to first-level referral hospitals, a higher proportion of tertiary hospitals had basic sanitation for NCUs (60% vs 40% for first-level referral hospitals, *P* = 0.53). Tertiary hospitals were also more likely to have clean toilets next to NCUs (40% vs 20% for first-level referral hospitals, *P* = 0.49) but less likely in or next to delivery rooms (40% vs 60% for first-level referral hospitals, *P* = 0.53).

#### Hygienic practices at childbirth

Birth attendants washed their hands before touching any delivery areas or surfaces in 78% (n = 289) and before gloving for delivery in 81% (n = 300) of 371 observed deliveries. The umbilical cord was hygienically handled with sterile gloves in 84% (n = 309) of deliveries. Appropriate hygiene (all three actions) was practiced in 65% (n = 240) of deliveries. Washing of hands before touching any delivery surfaces was practiced in fewer deliveries in tertiary hospitals (71% vs 83% for first-level referral hospitals, *P* < 0.001), but handling of the umbilical cord with sterile gloves was practiced in more deliveries at tertiary hospitals (89% vs 80% for first-level referral hospitals, *P* = 0.006).

Appropriate hand hygiene was practiced in all deliveries observed in a higher proportion of hospitals where all delivery rooms had a sink with water and soap compared to hospitals where this was not available in all rooms (50% vs 39%, *P* = 0.29).

## DISCUSSION

This study found that mothers and babies in seven countries in East Asia and the Pacific are often exposed to an elevated risk of infection due to insufficient hospital WASH services and gaps in hygiene practices at childbirth. Only 71% of hospitals provided a basic water supply in the delivery room, 71% in the NCU, and 32% in postnatal care rooms. Basic hygiene services (sink with water and soap or alcohol hand gel) was available in the delivery room of 77%, in the NCU of 78%, and in postnatal care rooms in 42% of hospitals assessed. These percentages declined to 36%-73% of hospitals for adequate hygiene services (all sinks are clean, with water, soap, and a hand drying method or alcohol hand gel is available). In analyses of data from 10 hospitals in Lao PDR and Papua New Guinea, basic sanitation was available in or next to delivery rooms in 60% of hospitals and next to NCUs in 50% of hospitals. The figure declined to 50% for delivery rooms and 30% for NCUs when availability of basic and clean sanitation facilities was assessed. These findings are consistent with previous reports from within and outside the region on availability of WASH services, suggesting that this remains a pervasive problem [7,16,17].

Supportive policies and standards are important to ensuring service provision in health facilities [7]. Of the seven countries included here, three had national policies and three national standards on WASH in health facilities. Standards were associated with availability of piped water supply and all hand hygiene services. Policies were not significantly associated with increased availability of any WASH service, suggesting that in these countries, standards may be more important for guiding action at the facility level. Further investigation is required to examine possible reasons why national policies are not linked to facility-level change and whether this observation holds true more widely.

Other factors contribute to inadequate WASH services including lack of monitoring, limited knowledge and awareness of health facility staff, lack of space and costs of infrastructure changes [7]. Postnatal care areas are most likely to lack hygiene resources, despite the fact that these often receive large numbers of family visitors all of whom should practice hand hygiene before and after touching newborns. Responsibility for provision of soap and towels often rests with families who may not be able to afford supplies or lack awareness of their importance. Even when hand hygiene resources are available, family members are often not instructed on hand hygiene practices. The pervasive use of mobile phones, particularly in postnatal care areas, potentially compounds the problem of infection risk [18].

We also found poor adherence to hygiene during childbirth. In one in three deliveries, birth attendants did not wash hands prior to touching delivery area surfaces and equipment and immediately prior to gloving for delivery, and handle the umbilical cord with uncontaminated sterile gloves. Similar findings have been reported in other settings [19,20]. Compliance with hand hygiene is influenced by several factors including practices of peers, perceptions of acuity of care and self-protection, knowledge, workload, and availability of gloves and hand hygiene resources [21]. We found that appropriate hygiene was more likely to be practiced when sinks with water and soap were available in delivery rooms (albeit with large room for improvement).

Insufficient WASH services and compliance to hygiene have important implications for maternal and newborn health. Inadequate sanitation facilities have been significantly associated with higher maternal mortality [22], and poor hygiene with neonatal mortality [3,5]. Health worker hand hygiene is the most important iatrogenic factor associated with hospital acquired neonatal infections [23]. In an analysis of 85 infection outbreaks in neonatal nurseries in 1990-2004, infected hands of staff were reported as a common source of infection, with a lack of water for handwashing identified as a reason [5].

Without quality facility-based delivery care, impact on mortality is limited compared to home care [24]. Perceived quality of care is also an important factor driving decisions to deliver in facilities and follow the guidance of health care providers [25-28]. Thus, universal WASH services in health facilities are critical to accelerating reductions in preventable mortality.

Improving WASH services requires action at the national and health facility levels. Greater commitment is needed to develop, endorse, and implement policies and standards, which specify requirements for different services including childbirth and newborn care. Monitoring implementation is particularly critical as translation of policies and standards into service delivery in health facilities remains a challenge in many settings, as suggested by findings from this study. The JMP is currently developing indicators on monitoring WASH and IPC practices in delivery rooms, which in the longer term will be integrated into facility information systems and reported routinely. Countries like Ethiopia, India and Viet Nam have rolled out national initiatives to improve cleanliness, hygiene and overall IPC in health facilities [29-31], however the impact of these has yet to be evaluated.

At the health facility level, quality improvement approaches are being promoted to improve WASH and IPC. The quality improvement tool adopted by countries implementing EENC [12] has been regularly used by hospitals for self-monitoring and action to improve quality of maternal and newborn care, including WASH services. Actions taken to address identified gaps have included improving availability of hand washing materials, re-organization of delivery room environments to allow more time for adequate preparation, and coaching delivery attendants on appropriate hand hygiene [11,15]. Through implementation of EENC and the quality improvement approach, a regional hospital in Viet Nam has reported significant reductions in NCU admissions, and hypothermia and newborn sepsis rates [11].

This study was limited to 147 hospitals, representing around 12% of hospitals providing childbirth services in the seven countries with at least 50 deliveries per year [15]. Findings are therefore not representative of all hospitals and cannot be generalized to lower level facilities or home births attended by skilled birth attendants. Data on sanitation facilities was available from only two countries and therefore cannot be considered representative of hospitals in the region. These findings suggest that sanitation data are often not collected routinely in most countries. In the seven selected countries, the proportion of institutional births delivered in hospitals ranges between 50% and over 90%, making them an important provider of delivery care in all countries. Facilities that have begun implementation of EENC may be more likely to be performing better than those that have not begun implementation. In addition, coverage of WASH services is more likely to be better at tertiary hospitals and first level referral hospitals than at lower level facilities. For these reasons, we believe the findings presented here overestimate the quality of facility WASH services more generally.

The cross-sectional design of the study represents a snapshot of WASH services that may vary over time for several reasons including the availability of financial and other resources. Observation bias was possible when hand washing and sanitation facilities were assessed for functionality and cleanliness. Validation of assessment findings was not conducted to assess the internal validity of observations. Since all national assessments were conducted using the same methodology, standard training for surveyors, identical data collection tools and indicator definitions, overseen by external supervisors, we believe this problem to be minimal. Sanitation data were collected from self-monitoring assessments conducted by hospital teams also using standard methods, tools and indicator definitions, but without external supervision and may therefore be more likely to be subject to bias. However, within countries, assessments did not show significant variations between hospitals of the same category that might suggest systematic observer bias. We also did not assess factors that affect quality of services, such as continuity of services over time, availability of sufficient quantities of uncontaminated water, and broader aspects of sanitation such as methods of wastewater treatment and overall cleanliness of hospitals, all important for patient safety and comfort.

Observations of delivery practices were limited to deliveries conducted at the time of assessments. Quality of delivery care may change with case-load, case complexity, staffing patterns and time of the day. Observations were conducted by observers trained in EENC clinical practice, using standard checklists to limit potential observation bias. Observations of delivery practice may have also been subject to the Hawthorne effect resulting in a bias to improved practices. The consistent finding of hygiene practice gaps however, suggests that observation bias did not significantly influence practices reported here.

## CONCLUSIONS

WASH is emphasized in Sustainable Development Goal targets 6.1 and 6.2 [32] as well as the Global Strategy for Women's, Children's and Adolescent's Health [33] and Standards for improving quality of maternal and newborn care in health facilities [14]. Our findings show that WASH services for maternal and newborn care remain inadequate in hospitals in East Asia and the Pacific. All countries should adopt WASH standards aligned with international recommendations and incorporate WASH indicators into routine monitoring systems. Assessments to determine the availability of services in facilities at all levels are needed, along with studies to better determine correlations between WASH service availability, hygiene practices and infectious disease outcomes. Regular monitoring is essential for tracking progress and should be integrated into approaches to improving quality of care. The EENC approach, adopted in the seven countries assessed here, does so using regular hospital self-monitoring by hospital teams and shows promise for future progress. Addressing hygiene and sanitation limitations requires not just improvements in availability of infrastructure and supplies but also changes in work environments needed to motivate staff to consistently apply appropriate WASH practices.
